# The Role of Iron and Copper on the Oligomerization Dynamics of DR_2577, the Main S-Layer Protein of *Deinococcus radiodurans*

**DOI:** 10.3389/fmicb.2019.01450

**Published:** 2019-06-28

**Authors:** Domenica Farci, Giulia Guadalupi, Katarzyna Bierła, Ryszard Lobinski, Dario Piano

**Affiliations:** ^1^ Department of Plant Physiology, Warsaw University of Life Sciences - SGGW, Warsaw, Poland; ^2^ Laboratory of Photobiology and Plant Physiology, Department of Life and Environmental Sciences University of Cagliari, Cagliari, Italy; ^3^ Laboratory of Analitycal and Bioinorganic Chemistry and Environment, UMR5254 Institute of Analytical and Physical Chemistry for the Environment and Materials (IPREM), Pau, France

**Keywords:** *Deinococcus radiodurans*, DR_2577, metal-binding domain, oligomerization, SlpA, S-layer deinoxanthin binding complex

## Abstract

Surface (S)-layers are cryptic structures that coat the external surface of the bacterial cell in many species. The paracrystalline regularity of the S-layer is due to the self-assembling of one or more protein units. The property of self-assembling seems to be mediated by specific topologies of the S-layer proteins as well as the presence of specific ions that provide support in building and stabilizing the bi-dimensional S-layer organization. In the present study, we have investigated the self-assembling mechanism of the main S-layer protein of *Deinococcus radiodurans* (DR_2577) finding an unusual role played by Fe^3+^ and Cu^2+^ in the oligomerization of this protein. These findings may trace a structural and functional metallo-mediated convergence between the role of these metals in the assembling of the S-layer and their well-known roles in protecting against oxidative stress in *D. radiodurans*.

## Introduction

*Deinococcus radiodurans* is a pink-pigmented extremophile well known for its ability to withstand high doses of ionizing and UV radiations, as well as deep desiccation (Mattimore and Battista, 1996; Battista, 1997; Battista et al., 1999; Cox and Battista, 2005; Fredrickson et al., 2008; Slade and Raman, 2011; Das and Misra, 2013; Farci et al., 2016a, 2018a). This bacterium has been extensively studied for its surface layer (S-layer), a cell structure characterized by a main protein complex named S-layer Deinoxanthin Binding Complex (SDBC). SDBC is a homo-hexamer of ~760 kDa and is composed of the S-layer protein DR_2577, also known as SlpA, to which is bound the carotenoid cofactor deinoxanthin (Farci et al., 2015, 2016a, 2017). We recently showed that this protein complex exhibits spectral properties and thermal stability that comply with the resistance to UV-radiation and deep desiccation (Farci et al., 2016a, 2018a).

The S-layer of *D. radiodurans* is a proteinaceous-paracrystalline array with a p6 (hexagonal) symmetry (Baumeister et al., 1982; Farci et al., 2014) that was shown to be constituted by stable dimers of the protein DR_2577 further assembled into trimers of dimers, eventually composing the SDBC hexameric complexes (Farci et al., 2015). In spite of the extended biochemical characterization of the protein DR_2577, the mechanism of assembling into dimers and hexamers still remains unknown. We have performed preliminary bioinformatic analyses that predicted, with good confidence, the presence of an iron-binding domain. Experimental studies confirmed the predicted presence of iron and also identified the presence of copper. Accordingly, we have performed specific *in gel* iron- and copper-induced oligomerization studies at different pHs. The results shown here confirm a role played by iron and copper in the DR_2577 oligomerization mechanism and are in agreement with the role of pH and polyvalent cations on the assembly and turnover of other bacterial S-layers (Chester and Muray, 1978; Messner et al., 1986; Berenguer et al., 1988; Walker et al., 1992; Yang et al., 1992; Walker and Smit, 1993; Garduño et al., 1995; Pum et al., 2013; Farci et al., 2018b). Moreover, the presence of iron and copper in the DR_2577, and therefore in the cell wall of *D. radiodurans*, is in line with previous observations identifying the role of metals and their ratios in providing either ROS-resistance or ROS-sensitivity in different microorganisms (Daly et al., 2004, 2007; Omelchenko et al., 2005; Daly, 2009). Implications of these findings are discussed on the basis of the existing model of the S-layer in this bacterium and the essential protective role played by DR_2577.

## Materials and Methods

### Bacterial Strain and Growth Conditions

Culture media tryptone/glucose/yeast extract broth (TGY; Murray, 1992) was used to grow *D. radiodurans* strain R1 (ATCC 13939) for 24 h at 30°C under shaking (250 rpm). Cells were harvested by centrifugation at 5,000*g* for 10 min at 4°C and resuspended with Buffer A (50 mM sodium phosphate pH 7.8).

### DR_2577 Enriched Membranes Preparation

Cell wall fragments were purified at 4°C according to Farci et al. (2014). Resuspended cells were treated with DNase and disrupted using a French Pressure Cell operating at 1,100 psi. A step of low speed centrifugation (4°C, 2 × 2,000*g* for 10 min) was used to remove non-lysed cells. The supernatant was then centrifuged at higher speed (4°C, 48,000*g* for 10 min), obtaining a pink pellet with the most homogeneous and small membranes fragments. This pellet was resuspended in 1.5 ml of Buffer A, containing 100 μg/ml lysozyme and incubated under agitation (200 rpm) for 8 h at 25°C. This step was aimed at removing surface polysaccharides. The membrane suspension was then centrifuged (4°C, 4 × 48,000*g* for 10 min) to separate the membranes from polysaccharides and lysozyme.

### Membranes Solubilization

The final pellet was resuspended, homogenized, and solubilized by agitation in 1% (w/v) n-dodecyl-β-D-maltoside (β-DDM) at room temperature for 1 h. The solution was then centrifuged (4°C, 48,000*g* for 10 min) to remove insoluble material.

### Anion-Exchange Chromatography

After solubilization and centrifugation, the supernatant obtained was loaded in a Q Sepharose high performance anion-exchange chromatography column (GE Healthcare, Chicago, USA) previously equilibrated with Buffer B [50 mM Na phosphate pH 7.4, 0.05% (w/v) β-DDM] at a flow rate of 0.5 ml/min. After injection, the column was washed with five column volumes of Buffer B (flow rate of 0.5 ml/min) until the absorbance was stable. Finally, the bound components were eluted in a gradient of 0–100% Buffer C [50 mM Na phosphate pH 7.4, 2.5 M NaCl, 0.05% (w/v) β-DDM].

### Size Exclusion Chromatography

The DR_2577 fractions obtained from the anion-exchange chromatography were pooled together and concentrated reaching a final volume of 500 μl using a Vivaspin 20 (GE Healthcare, Chicago, USA) ultrafiltration membrane with 100 kDa MW cutoff. The concentrated sample was then loaded on a Superose 6 column (Superose 6 10/300GL, GE Healthcare) previously equilibrated in Buffer B [50 mM Na phosphate pH 7.4, 0.05% (w/v) β-DDM]. The equilibration and the run were performed at a flow rate of 0.5 ml/min.

### Inductively Coupled Plasma Mass Spectrometry

ICP-MS experiments were performed on an Agilent Model 7,500 ce (Agilent, Santa Clara, USA) equipped with an octopole reaction cell. Prior to ICP analysis, samples were diluted 1:10 with 0.2% formic acid and injected though a Micromist nebulizer (Glass Expansion, Romainmotier, Switzerland) fitted with a double-pass Scott spray chamber (2°C). ICP-MS measurement conditions (nebulizer gas flow, RF power, and lens voltage) were optimized by using a multi-element solution (Li, Y, and Tl). Iron detection was achieved after removing the interferences by using H_2_ reaction gas (octopole bias −18 V; quadrupole bias −16 V) in the octopole reaction cell. Iron and copper concentration analysis was carried out by measuring the intensities of all iron and copper isotopes with an integration time of 0.6 s per mass. Background intensities measured in blank solution were subtracted from sample intensities. Total iron and copper concentrations were calculated using an external calibration curve at four levels.

Analytical reagent grade chemicals purchased from Sigma-Aldrich (Saint-Quentin Fallavier, France) and water (18.2 mega ohm cm) obtained with a Milli-Q system (Millipore, Bedford, MA) were used throughout unless stated otherwise. The Fe and Cu standard solutions were obtained from SPC Science (Quebec, Canada).

### Bioinformatic Analysis

DR_2577 sequence was taken from the Universal Protein Knowledgebase (UniProt) server (http://www.uniprot.org/; Apweiler et al., 2004; The Uniprot Consortium, 2019). The pI was calculated by using the Compute pI/MW tool from the ExPASy server (https://web.expasy.org/compute_pi/; Gasteiger et al., 2005). The binding site prediction for metals was done by using the RaptorX – Binding (http://raptorx.uchicago.edu/BindingSite/; Källberg et al., 2012).

### Polyacrylamide Gel Electrophoresis

Sodium Dodecyl Sulfate-Polyacrylamide Gel Electrophoresis (SDS-PAGE) was performed with a 7% (w/v) resolving polyacrylamide/urea gel and a 4% (w/v) stacking gel (Farci et al., 2015, 2016b). DR_2577 samples were denatured with SDS in the presence of β-mercaptoethanol by using Roti-Load 1 (Carl Roth, pH 6.6–7.2). However, samples were not boiled in order to 1) avoid any interference with the oligomerization process and 2) not induce monomerization. After the electrophoretic separation, the gels were fixed and stained with Coomassie Brilliant Blue G250 (Serva, Germany) for 2 h and destained for 1 h with a destaining solution (7% acetic acid, 10% ethanol).

### Oligomerization and Monomerization Tests on Sodium Dodecyl Sulfate-Polyacrylamide Gel Electrophoresis and Size Exclusion Chromatography

Experiments were designed considering (1) the metal identification assay by ICP-MS described in section “Inductively Coupled Plasma Mass Spectrometry,” (2) the bioinformatic prediction on the presence of metal-binding sites associated to the protein described in section “Bioinformatic Analysis,” and (3) previous observations about the primary role of metals in this bacterium made by Daly et al. (2004, 2007) and Daly, 2009. Oligomerization tests were performed on 10 μl of protein at 0.2 mg/ml and Cu^2+^ and Fe^3+^ effects were investigated with a final concentration range spanning between 10 and 20 mM starting from 150 mM stock solutions of CuSO_4_ or Fe_2_(SO_4_)_3_. Controls for monomerization assays were performed by metal chelation using ethylenediaminetetraacetic acid (EDTA; 1 M stock). The effect of pH on monomerization was tested in the presence of Na phosphate (50 mM) at different pHs (7.0, 6.0, 5.0, 4.0). Grid screen of different metals (Cu^2+^, Fe^3+^, and Ca^2+^) at concentrations from 5 to 15 mM versus pHs 6.0 and 7.0 was tested according to the sample processing, protein concentration, and amounts described above. The obtained mixes of each sample were incubated for 10 min at 25°C and, subsequently, processed for SDS-PAGE analysis. The oligomerization/monomerization effect was assessed using a control without/with EDTA or metal ions.

Equivalent assays were analyzed under close-to-native conditions by SEC. These experiments were performed on 50 μl protein at 0.2 mg/ml previously incubated according to the conditions described above. Each run was performed according to the description in section “Size Exclusion Chromatography,” but for each condition tested, the SEC buffer was modified accordingly. In particular, for pH ≤ 6, a 50 mM Na acetate buffer was used, while for pH ≥ 7, a 50 mM phosphate buffer was used. In both cases, buffers were added with 0.05% (w/v) β-DDM, while EDTA and/or metals were added according to the condition tested.

## Results

### Bioinformatic Analysis Predicted a Fe^3+^ Binding Site

**Figure 1:**
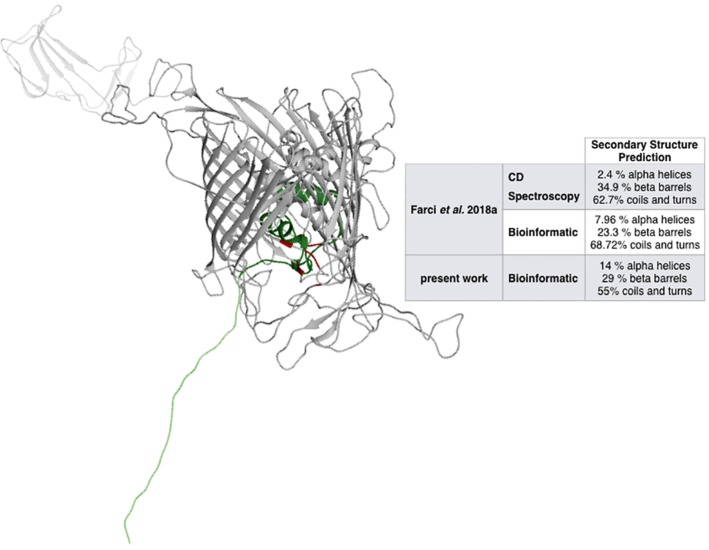
Structural prediction of DR_2577. The inset compares the bioinformatic analysis made by RaptorX with the analyses previously reported (Farci et al., 2018a). In red, the Fe-binding domain is indicated, while the dark and light green regions indicate the Slh and the transmembrane domains, respectively.

Metal ions are expected to play an active role in the assembling and the oligomerization of a large variety of S-layer proteins, including the S-layer proteins of species phylogenetically related to *D. radiodurans* such as *Thermus thermophilus* (Berenguer et al., 1988; Sára and Sleytr, 2000; Farci et al., 2018b). While these species have remarkably different phenotypes (Omelchenko et al., 2005), they maintain a significant degree of similarity between their main S-layer proteins (Rothfuss et al., 2006). Accordingly, the RaptorX – Binding software was used to identify potential cation-binding sites (Table 1) and to predict the DR_2577 structure (Figure 1) on the basis of the representativeness of secondary structures (Figure 1, inset). The relative relevance of each secondary domain obtained from the bioinformatic prediction was found to be in agreement with previous reports for DR_2577 (Farci et al., 2018a), and with typical β-barrel structures of porins (Seshadri et al., 1998; Fairman et al., 2011) as well as of retinol-binding proteins (Newcomer and Ong, 2000; Zanotti and Berni, 2004). Surprisingly, this bioinformatic analysis predicted the presence of a Fe^3+^-binding domain with high confidence, while were not predicted domains for typical S-layer binding metals, such as Ca^2+^ and Mg^2+^. This domain was identified into a pocket defined by residues A484, T485 T486, N493, P494, I496, G529, S530, N531, and D684 (Table 1, Figure 1).

**Table 1:** Metal binding prediction.

Ligand	Residues	Score^*^
Fe^3+^	A484, T485 T486, N493, P494, I496, G529, S530, N531, D684	49

* *Values >40 are considered reliable*.

### DR_2577 Binds Cu and Fe

Next, the presence of metals was assessed by an inductively coupled plasma mass spectrometry (ICP-MS) assay on DR_2577 samples, as reported by Becker et al. (2008). Measurements were done on the purified protein in solution using the protein buffer (Buffer B: phosphate buffer 50 mM, pH 7.4, 0.05% β-DDM) as a blank. These experiments confirmed the presence of Fe in the samples and led to the unpredicted detection of significant amounts of Cu. In particular, Cu was present in higher amounts (263.85 ± 5.72 ng/ml) with respect to Fe (96.50 ± 11.66 ng/ml), finding a ratio between the two metals of about 3:1 (Table 2).

**Table 2:** Metal quantification by ICP analysis.

	Fe (ng/ml)	Cu (ng/ml)
Buffer (blank)	26.56 ± 2.9	12.00 ± 0.61
DR_2577 (sample)	96.50 ± 11.66	263.85 ± 5.72

### Metal Presence Is Further Confirmed by Ethylenediaminetetraacetic Acid-Induced Monomerization

**Figure 2:**
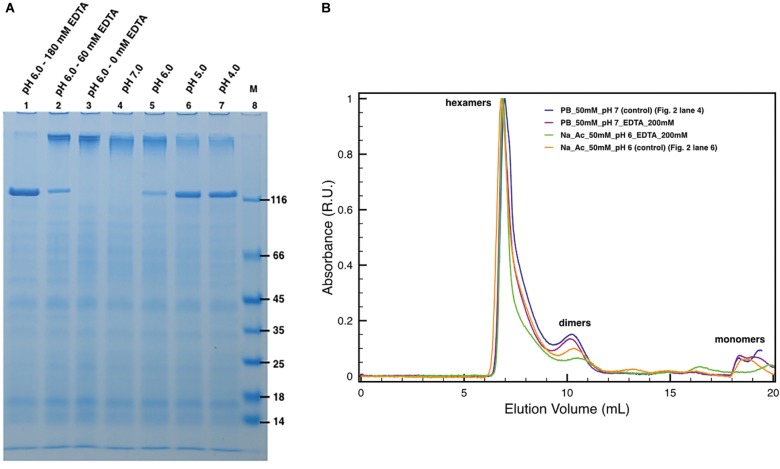
Induced monomerization by pH and EDTA. **(A)** Lanes 1–3 show the effect of different EDTA concentrations under subacid conditions (pH 6.0). Lanes from 4 to 7 show the monomerization by the only mean of pH at pH 7.0, 6.0, 5.0, and 4.0, respectively. In lane 8, (M) is the molecular marker. Samples in lanes 2 and 3 were diluted with 50 mM buffer phosphate at pH 7.8 to compensate the missing volume of EDTA. Dilutions related to samples processing led to small pH increases explaining the differences between lanes 3 and 5; **(B)** plots with SEC runs under conditions equivalent to the ones shown in SDS-PAGEs (for details, see legend in the picture).

The protein DR_2577 tends to form very stable dimers (Farci et al., 2015). As also observed in other S-layer proteins, oligomerization properties are usually mediated by bivalent cations, such as Ca^2+^ and Mg^2+^ (Pum et al., 2013), and monomerization can be obtained by EDTA treatment, as also shown for the DR_2577 homologous in *T. thermophilus* (Farci et al., 2018b). In order to see whether Cu and Fe may play a similar role in the DR_2577 oligomerization, experiments of EDTA-induced monomerization were performed and analyzed by SDS-PAGE and SEC. SDS-PAGE experiments clearly showed a concentration-dependent monomerization effect induced by EDTA proving not only evidence for the presence of metals, but also indicating their role in the protein oligomerization (Figure 2A, lanes 1–3). On the contrary, when DR_2577 samples were studied by SEC, the concomitant absence of monomers and a limited amount of dimers, the latest known to be the DR_2577 building blocks stabilized by intermolecular disulfide bonds (Farci et al., 2015), were associated with the persistence of a peak at higher masses ascribable to DR_2577 hexamers (Figure 2B). Moreover, the variable intensity of the second peak is due to a partial monomerization (and possible degradation) especially associated to lower pHs and the presence of EDTA (this also in agreement with the results of Figure 2A). This finding, obtained under close-to-native conditions, provides an evidence for a DR_2577 oligomerization mediated not only by metal bridges between dimers but also by other intermolecular interactions.

Considering that these properties are dependent from metals carrying two different oxidation states, also a functional role, related to the protective properties of DR_2577 (Farci et al., 2016a), might be coexistent with the structural one related to the oligomerization.

### DR_2577 Monomerization Is Influenced by pH

Next, specific assays were conducted by SDS-PAGE and SEC to probe the DR_2577 monomerization at different pHs (Figure 2). These experiments showed that in the conditions where the DR_2577 protein has a positive net charge, i.e., near or below pH 5 (DR_2577 has a theoretical pI 5.0), its monomerization is induced partially (pH 6.0) or completely (pH 5.0 and 4.0) even in the absence of EDTA (Figure 2A, lanes 4–7). Furthermore, as shown in the previous paragraph, physiological subacid environments (pH 6.0) are able to induce monomerization proportionally to the EDTA provided (Figure 2A, lanes 1–3). The absence of monomerization under neutral conditions (Figure 2A, lane 4) showed that the DR_2577 oligomerization takes place through metals different than Mg^2+^ and Ca^2+^, which on the contrary are reported to be efficiently chelated by EDTA in neutral conditions (Berenguer et al., 1988; Farci et al., 2018b). This differs from what observed for the main S-layer protein of the phylogenetically related (but phenotypically unrelated) bacterium *T. thermophilus* (Berenguer et al., 1988; Farci et al., 2018b) and other species (Chester and Muray, 1978; Walker et al., 1992; Yang et al., 1992; Walker and Smit, 1993; Garduño et al., 1995). As observed in the previous paragraph, also in these series of experiments, no effects of monomerization could be observed when the samples were studied by SEC (Figure 2B), further suggesting that in the oligomerization of DR_2577 might be essential not only the metal bridges but also other intermolecular interactions.

Reassembling experiments were also performed. They confirmed the evidence of the role played by pH and low concentrations of metals for inducing oligomer formation starting from monomers. However, these experiments showed to be poorly reproducible suggesting the presence of also other factors involved in the reassembling and thus in the regulation of the DR_2577 assembling (data not shown).

### DR_2577 Monomerization Is Also Induced by Cu^2+^ and Fe^3+^

**Figure 3:**
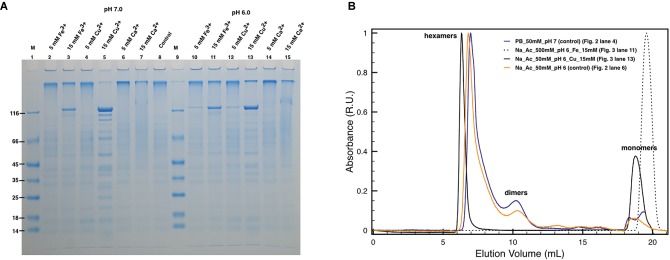
Effect of metals on DR_2577 monomerization. **(A)** Cu^2+^, Fe^3+^, and Ca^2+^ were tested at concentrations of 5 and 15 mM under pH 7.0 (lanes 2, 3, 4, 5, 6, 7, respectively) and under pH 6.0 (lanes 10, 11, 12, 13, 14, 15, respectively). Lanes 1 and 9 (M) are the molecular marker; lane 8 is the control consisting in DR_2577 at pH 7.8 and in the absence of metals; **(B)** plot with SEC runs under conditions equivalent to the ones shown in SDS-PAGEs (for details, see legend in the picture).

Subsequently, similar experiments in the absence of EDTA allowed to analyze the possible role of Cu and Fe in the protein oligomerization (Figure 3) considering the influence of the pH on their chelation (Wu et al., 1999; Albano and Miller, 2001). Unexpectedly, concentrations of Cu^2+^ and Fe^3+^ above 15 mM appeared to also induce DR_2577 monomerization at neutral (Figure 3A, lanes 3 and 5) and subacid (Figure 3A, lanes 11 and 13) pHs. These effects were almost pH independent, as observed in monomerization assays of DR_2577 samples at neutral and subacid pHs (7.0 and 6.0) in the absence of metals, where monomerization is minimal (Figure 2A, lanes 4 and 5; Figure 3A, lane 8). Interestingly, experiments performed using Cu^2+^, which was observed to be more efficient in inducing the monomerization with respect to Fe^3+^ in PAGEs, in SEC led to several important evidence: (1) changes in the high oligomers types leading to a more symmetric peak at slightly higher apparent masses; (2) the appearance of a significant amount of monomers with (3) a concomitant decrease of the dimers (Figure 3B). On the contrary, the use of Fe^3+^ showed an apparently lower monomerization efficiency in PAGEs, while on SEC experiments led to the exclusive formation of monomers. These differences suggested that the two metals *in vivo* exert a different and opposite effect in the S-layer: while Fe^3+^ promotes monomer formation, Cu^2+^ induces this effect much less efficiently and stabilizes oligomers.

In order to understand the degree of specificity with respect to this effect, we also tested the effect of a bivalent metal such as Ca^2+^, which is known to play an important role in the assembling of other S-layers at neutral pH. However, this cation did not induce any significative monomerization either at neutral (Figure 3A, lanes 6 and 7) or at subacid pH (Figure 3A, lanes 14 and 15).

## Discussion

In spite of the very well-known description of the top organization of S-layers, a deep lack of knowledge still exists with respect to their structural properties and three dimensional organizations, which have hardly been reported (Bharat et al., 2017). There is also a lack of information with respect to the functional roles played by S-layers, which alone represent 10–15% of the total protein fraction of the bacterial cell, providing an objective proof of their primary role (Sleytr et al., 1993).

Specifically, the S-layer of *D. radiodurans* has been studied for its interesting functional traits provided by the presence of the carotenoid deinoxanthin associated to its main protein DR_2577 (Farci et al., 2016a, 2017). This S-layer protein, through its cofactor, allows the S-layer to behave as a shield against UV stress and desiccation (Farci et al., 2016a).

With the final aim to understand the molecular mechanisms by which this S-layer performs its protection, we have investigated the possible role of metal ions on its structural and functional properties. The presence of metals in S-layers and their role in stability and assembling were extensively reported (Chester and Muray, 1978; Messner et al., 1986; Berenguer et al., 1988; Walker et al., 1992; Yang et al., 1992; Walker and Smit, 1993; Garduño et al., 1995; Pum et al., 2013; Farci et al., 2018b), and they appear to be typically limited to cations with a single oxidation state such as Ca^2+^ and Mg^2+^ (Pum et al., 2013). Starting from the bioinformatic prediction (Table 1) and subsequent experimental studies (Table 2), we have identified the presence of Cu and Fe in the main S-layer protein of *D. radiodurans*, DR_2577. This protein binds both metals (Table 1), as confirmed by the ICP-MS analysis (Table 2), and the carotenoid deinoxanthin (Farci et al., 2015); these properties would be integrated in the 22 strands β-barrel predicted structure (Figure 1), which is typical for porins (Seshadri et al., 1998; Fairman et al., 2011), but also diffused, even if with a reduced strand number, in retinol-binding proteins (Newcomer and Ong, 2000; Zanotti and Berni, 2004).

Further experiments were aimed at understanding the possible structural and functional role played by Cu^2+^ and Fe^3+^ on this specific S-layer. Accordingly, metal chelation experiments showed how these metals may have a functional role in the oligomerization properties (Figure 2A, lanes 1–3) and the related stability of the S-layer, as previously observed for Ca^2+^ and Mg^2+^ in other S-layers (Chester and Muray, 1978; Messner et al., 1986; Berenguer et al., 1988; Walker et al., 1992; Yang et al., 1992; Walker and Smit, 1993; Garduño et al., 1995; Pum et al., 2013; Farci et al., 2018b).

In *D. radiodurans*, Fe, Cu, and Mn were reported to be involved in mechanisms of resistance to oxidative stress (Daly et al., 2004, 2007; Daly, 2009). In particular, a protective mechanism in *D. radiodurans* was attributed to the presence of high levels of Mn^2+^ and low levels of Fe^2+^ (Daly et al., 2004). Indeed, the release of Fe^2+^ from proteins out to the cell would reduce the impact of Fenton chemistry into the cells (Daly, 2009). In this context, the predicted β-barrel structures of DR_2577 typical for porins and the high levels of Fe^2+^ detected in its samples suggest a role of DR_2577 in gating cations and in particular Cu^2+^ and Fe^2+^ out from the cell. Furthermore, Cu and Fe are well known to be crucial metal cofactors of enzymes responsible for detoxication, such as in the mechanisms of reactive oxygen species (ROS) scavenging mediated by the enzymes catalase and superoxide dismutase (Lu et al., 2017). This fact suggests that, in addition to the structural stabilization role on the S-layer, these cations may also have an active role in the protective function already demonstrated for this S-layer (Farci et al., 2016a, 2018a). In fact, they could take part in detoxication mechanisms from harmful ROS, which are formed during the “shielding” activity specific of this S-layer. Notably, between the photo- and the chemo-protective mechanisms hypothesized for the shielding properties of DR_2577 against UV light (Farci et al., 2016a, 2018a), the presence of these cations strongly corroborates the latter hypothesis. This mechanism may involve a UV induced cationic form of deinoxanthin that can behave as an antioxidant against environmental ROS and that is most likely quenched through reducing agents provided by the cell and mediated by metals. In this respect, the presence of DR_2577 orthologs in ROS- and UV-sensitive species, such as *T. thermophilus* (Berenguer et al., 1988; Farci et al., 2018b), supports the idea of the S-layer proteins as structures that share very robust scaffolds with common surface properties (e.g., bidimensional self-assembling, metal and sugar binding properties), but for which the evolution shaped peculiar functionalizations imposed by specific environments.

An important role in the DR_2577 monomerization is evidently played by the pH. In general, pH values below 6.0 are able to induce protein monomerization in SDS-PAGEs (Figure 2A, lanes 4–7) but do not show significant changes in SEC with respect to control runs (Figure 2B). The monomerization at low pH observed on SDS-PAGEs (Figure 2A, lanes 4–7) may be explained by considering that DR_2577 has a pI of 5.0. This implies that at pH < 5.0 the protein assumes a net positive charge becoming less affine to cations; thus, the monomerization is promoted. However, this effect cannot be considered as the only one responsible for monomers assembling/disassembling. In fact, the absence of monomers in native samples treated under low pH (Figure 2B) strongly suggests that also other forces independent from pH are involved (e.g., hydrophobic interactions). In every case, the effect of low pH on the DR_2577 oligomerization may also be seen as a functional mechanism through which the S-layer switches from an active (metal binding) to an inactive (metal free) form. Neutral and subacid pHs (pH 7–6) were shown not to induce significant monomerization; thus, they are ideal for assessing the monomerization induced by Cu^2+^ and Fe^3+^. Under these pH conditions, 15 mM of either Cu^2+^ or Fe^3+^ is able to induce monomerization as observed on SDS-PAGEs (Figure 3A). However, in experiments performed under native conditions, while Fe^3+^ strongly confirmed the SDS-PAGEs results, Cu^2+^ only showed a minimal monomerization effect (Figure 3B). This apparent discrepancy may be explained in terms of different forces involved in the formation of DR_2577 oligomers, as previously mentioned. In this case, Fe^3+^ induced the complete removal of whatever type of oligomers, inducing the formation of monomers (Figure 3B). Contrariwise, Cu^2+^ promoted the appearance of both a nicely symmetric peak at slightly higher apparent masses, suggesting the formation of homogeneous hexamers, and of a small amount of monomers, resulting from the removal of intermediate and/or incomplete oligomeric states (Figure 3B). Results can be explained according to a model where Cu^2+^ is involved in the inter-dimeric interactions, promoting the formation and stability of hexamers and the removal of all the incomplete intermediates, which can be observed in the control. By this mechanism, it would be possible to deliver monomers and dimers where the latest will take part in the hexamer formation. It must be pointed out that also on SDS-PAGE, the Cu^2+^ test is not able to induce full monomerization showing a sharpened band at heavy masses (Figure 3A, lane 13). This observation is in agreement with the SEC profile at the same conditions (Figure 3B).

On the contrary, the Fe^3+^ seems to directly act on dimers. Dimers are the basic units for DR_2577 oligomerization, and they were found to be stabilized by two intermolecular disulfide bonds (Farci et al., 2015). In this context, Fe^3+^ at the experimental concentrations used (15 mM) could act promoting the reduction of the disulfide bond, inducing the formation of monomers from dimers. This would explain the dominance of a single monomer peak in SEC experiments (Figure 3B) and a single monomeric band in SDS-PAGE experiments (Figure 3A).

In conclusion, the presented findings contribute to implement our understanding of the properties associated to the main S-layer protein DR_2577, indicating Cu and Fe as new important players having their role at the interface between the S-layer’s structure and function.

## Data Availability

All datasets generated for this study are included in the manuscript and/or the supplementary files.

## Author Contributions

DP and DF conceived, designed, and coordinated the study, carried out the membranes preparation, protein isolation, biochemical and bioinformatic studies, and drafted the manuscript. GG participated in the membranes preparation, participated in the protein isolation and biochemical studies, and helped in drafting the manuscript. RL and KB performed the ICP-MS analysis and helped in drafting the manuscript.

### Conflict of Interest Statement

The authors declare that the research was conducted in the absence of any commercial or financial relationships that could be construed as a potential conflict of interest.
